# Association of Righ-Risk Human Papillomavirus and *Ureaplasma parvum* Co-Infections with Increased Risk of Low-Grade Squamous Intraepithelial Cervical Lesions

**DOI:** 10.31557/APJCP.2021.22.4.1239

**Published:** 2021-04

**Authors:** Isabella Harumi Yonehara Noma, Cristiane Suemi Shinobu-Mesquita, Tamy Taianne Suehiro, Fabricio Morelli, Maria Vitória Felipe de Souza, Edilson Damke, Vânia Ramos Sela da Silva, Marcia Edilaine Lopes Consolaro

**Affiliations:** *Department of Clinical Analysis and Biomedicine, Clinical Cytology Laboratory, State University of Maringá (UEM), Paraná, Brazil. *

**Keywords:** Cervical cancer, high-risk HPV, Co-factors, mycoplasmas, risk

## Abstract

**Objective:**

The present report investigated the rates of coinfections between high-rik human papillomavirus (hrHPV) and the most important human mycoplasmas including *Mycoplasma hominis, M. genitalium, Ureaplasma urealyticum* and *U. parvum* in cervical samples of asymptomatic brazilian population.

**Methods:**

Were included a total of 283 women aged 25–64 years screened by Papanicolaou smears for determining cervical abnormalities, single-target polymerase chain reaction (PCR) and real-time PCR (rt-PCR) for hrHPV and mycoplasmas, respectively.

**Results:**

A total of 273 (94.5%) women were negative for intraepithelial lesions or malignancy cytology (NILM) and 10 (3.5%) presented abnormal cytology, all low-grade intraepithelial lesions (LSIL). The prevalence of hrHPV was 12.7% and 53.7% for mycoplasmas. *U. parvum* was the most frequently bacteria detected, followed by *Mycoplasma hominis* and *U. urealyticum. M. genitalium* was not detected. Women positive for *U. parvum* presented a 5-fold increased risk of LSIL (OR = 5.33; 95% CI = 1.09-26.04, P = 0.02) and co-infections between *U. parvum* and hrHPV increased the risk for LSIL (OR = 3.88; 95% CI = 1.75-8.58, P = 0.0003). However, these associations were not dependent on the concentration of the bacteria.

**Conclusion:**

Our results reinforced the hypothesis that some mycoplasmas may play a role as cofactors in HPV-mediated cervical carcinogenesis, at least in some populations.

## Introduction

Cervical cancer is the fourth leading cause of cancer in women worldwide, despite the existence of highly effective prevention and screening methods (Bray et al., 2018). Persistent high-risk human Papillomavirus (hrHPV) infection is the central factor in the development of squamous cell cervical carcinoma and hrHPV is a prerequisite for progression to high-grade squamous intraepithelial lesions (HSIL) (Kjaer et al., 2010). hrHPV infections are most eventually cleared, but few cases progress to HSIL (10% of HPV infections) and squamous cervical cancer (< 1% of HPV infections) (Ho et al., 1998; Tota et al., 2011; Monsego et al., 2015). The reasons for this variable natural history are poorly understood, but it is generally assumed that other causes or cofactors are important for the development of neoplasia in HPV-infected women (Bodily et al., 2011; Tota et al., 2011). The identification of these cofactors will improve the understanding of cervical carcinogenesis and will help to identify new opportunities for cervical cancer prevention and treatment (Adebamowo et al., 2017).

Several possible risk factors were associated with persistent hrHPV and contributed to cervical carcinogenesis (Moreno et al., 2002; Castle et al., 2003, Appleby et al., 2006; Carrillo-Gárcia et al., 2014). Among them, coinfection with sexually transmitted infections (STI) (de Abreu et al., 2016) as genital mycoplasmas, are gradually becoming more important (Ye et al., 2018). Genital mycoplasmas are groups of small free-living pathogenic bacterium belonging to class Mollicutes that lives on the ciliated epithelial cells of the urinary and genital tracts in humans (Tully et al., 1981), generally transmitted through direct interaction between hosts—venereally through genito-genital or oro-genital contact and vertically from mother to child (either in utero or at birth) (Taylor-Robinson, 2017). Genital mycoplasmas often refer to six species that colonize the genital tract, involving *Ureaplasma urealyticum*, *U. parvum*, *Mycoplasma hominis, M. genitalium, M. primatum*, and *M. spermatophilum. M. primatum and M. spermatophilum* are considered non-pathogenic for humans (Ljubin-Sternak and Mestrovic, 2014). Mycoplasmas have been associated with some asymptomatic and symptomatic genital tract conditions, including cervicitis, bacterial vaginosis and endometritis. Cervicitis, an acute or chronic inflammation of the uterine cervix, may promote the infection by human immunodeficiency virus and other STIs, including HPV, by the disruption of epithelial barrier integrity, and subsequent recruitment of inflammatory cells (Patel and Nyirjesky, 2010; Herfs et al., 2011; Silva et al., 2014). However, the results of previous studies on the association between prevalent mycoplasmas and hrHPV infections have been contradictory (biernat-Sudolska et al., 2011; Magaña-Contreraas et al., 2015; de Abreu et al., 2016; Kim et al., 2016; Klein et al., 2020). Therefore, it is still under discussion whether genital mycoplasmas may be a cofactor associated with hrHPV in cervical carcinogenesis.

The real prevalence of co-infections between hrHPV and mycoplasmas in cervical samples is not clear. The overall prevalence of genital mycoplasmas is an under-recognized condition in several populations despite its clinical importance, which is likely because of difficulties in the diagnoses that depend on traditional methods, such as culture (Daley et al., 2014). The recent development and introduction of cultivation-independent molecular-based techniques, such as polymerase chain reaction (PCR), revolutionized the diagnosis of genital tract infections (DÍnzeo et al., 2017) including mycoplasmas. Although studies have investigated cervical infections in Brazilian women, to date, few studies evaluated the coinfections between hrHPV and mycoplasmas in the Brazilian female population. Therefore, the present report investigated the rates of coinfections between hrHPV and the most important human mycoplasmas including *M. hominis, M. genitalium, U. urealyticum* and *U. parvum* in cervical samples of asymptomatic brazilian population. We found that *U. parvum* was the primary pathogen associated with hrHPV for increasing the risk of low-grade squamous intraepithelial lesions in this population, suggesting a possible synergistic action in cervical lesion development.

## Materials and Methods

*Study population*

This cross-sectional study enrolled asymptomatic women for STI and without a history of abnormal cytology residing in Maringá city/Brazil attending at the Public Health System to cervical cancer screening consult between March to October 2016. Women aged 25-64 years old were recruited because this is the recommended screening cohort by the Brazilian Cancer Institute. Women with any of the following factors were excluded: pregnancy, post-partum, previous hysterectomy, vaginal bleeding, previous history of cancer, sexual inactivity, recent treatment for any pathological condition of the urogenital tract, ablative or excisional therapy for the cervix within the previous 12 months, receiving treatment with antimicrobials (oral or topical) within the previous 4 weeks, and those using intrauterine devices or contraceptives delivered directly to the vaginal mucosa. Prior to undergoing gynecological examination, all women signed a written informed consent. Each woman voluntarily agreed to provide a sample of uterine cervix for Pap screening, hrHPV test and molecular mycoplasmas detection. This study was conducted with the supervision and approval of the Committee for Ethics in Research Involving Humans at the State University of Maringá (UEM)/Paraná/Brazil (CAAE: 37091114.2.0000.0104).

*Study procedures*

Demographic and baseline characteristics were obtained from analysis of the data from the standard registration form of each woman. Cytological samples were obtained from asymptomatic women for cervical cancer screening consult. Ecto/endocervical samples were collected with an Ayre’s spatula and cytobrush and were used for conventional Pap smear preparation. For hrHPV and mycoplasmas DNA analyses, samples were immediately suspended in liquid medium (HC2 DNA Collection Device; Digene, Gaithersburg, MD, USA) and stored at −10°C until analysis.

*Cytological diagnosis*

Cytological examination was conducted without the knowledge of hrHPV status at an accredited clinical laboratory (Clinical Cytology Laboratory of UEM) and reported according to the Bethesda 2001 nomenclature (Solomon and Nayar, 2015). The threshold of abnormal cytology was used to define all abnormal cytological findings including low-grade squamous intraepithelial lesions (LSIL), HSIL and cervical cancer. Negative for intraepithelial lesion or malignancy (NILM) was used to define normal cytological findings. 

*HPV detection and typing by Cobas HPV test*

Cervical specimens (2–5 mL of the liquid medium with sample) were tested for hrHPV DNA using the cobas HPV test (Roche Molecular Systems, USA) run on the fully automated cobas 4800 testing platform. The Cobas HPV test is a DNA test for hrHPV, and the results are reported in three separate channels: HPV16 individually, HPV18 individually, and a pool of 12 other hrHPV genotypes [11 definite high-risk, cancer-associated, HPV types (HPV 31, 33, 35, 39, 45, 51, 52, 56, 58, 59, and 68) plus one possibly hrHPV genotype (HPV66)]. A fourth channel measures β-globin for specimen adequacy. Inadequate specimens were retested and if still inadequate, results were treated as positive for patient safety purposes.

*Single-target polymerase chain reaction (sPCR) for mycoplasmas detection*

sPCR was conducted for qualitative detection of *Ureaplasma* spp., *M. hominis *and *M. genitalium*. DNA was extracted using the AxyPrep Body Fluid Miniprep Kit (Axygen, California, USA) according to the manufacturer’s instructions. The quality and quantity of the purified DNA were determined by spectrophotometry on a NanoDrop 2000 Spectrophotometer (Thermo Scientific, Massachusetts, USA).

For the design of primers (supplementary Table 1), specificity was checked against all sequences in GenBank and primers were aligned by using the Pairwise Sequence Alignment. Subsequently, the primers were evaluated by the OligoAnalyzer Tool (Integrated DNA Technologies) and selected according to melting temperatures, amplification temperature and amplicon size. All primers were evaluated performing a Basic Local Alignment Search Tool (BLAST) analysis against the sequences in the National Center for Biotechnology Information (NCBI) database. To assess the specificity of the primers, all primers were tested in sPCR reactions with different samples and positive control.

The optimized protocol for each assay was a mixture of 25 μL containing 2.5 mM of each deoxynucleotide triphosphates (dNTP), 1U of Taq DNA polymerase (Invitrogen, Carlsbad, CA), 0.6 mM of MgCl_2_, 25 mM of each primer and 50 ng of the extracted DNA for a final volume of 15 mL. The PCR conditions were comprised of thirty-five amplification cycles; denaturation for 10 min at 94ºC, annealing for 1 min at 55ºC, extension for 1 min at 72ºC, and final extension for 10 min at 72ºC (Thermal cycler, Biosystem,CA, USA). PCR products were electrophoresed on 1.0% agarose gel, stained with 1 mg/mL UniSafe Dye (Uniscience, USA), and photodocumented under UV light. Amplification of the human β-globin gene was performed as an internal control using primers GH20 and PC04. Positive controls for all studied bacteria were derived from positive clinical detected samples using reference methods (culture). Two types of controls were also included in each reaction, a “no-DNA” (negative control) and “positive DNA” for each bacteria (positive control). Samples positive for Ureaplasma spp. by sPCR were differentiated in U. urealyticum or *U. parvum* by real-time PCR as follows. 

*Real-time PCR (rt-PCR) assay for Ureaplasma spp. identification and mycoplasmas quantification*

rt-PCR was conducted for the identification of Ureaplasma spp. in *U. urealyticum* or *U. parvum* and for all mycoplasmas quantification using the standardized kits: Bio Gene Research Max *Mycoplasma* spp., Bio Gene Research Max *Ureaplasma parvum*, and Bio Gene Research Max *Ureaplasma urealyticum* (Bioclin, Brazil), following the manufacturer’s instructions. This assay has a detection range of 2 to 2X10^5^ copies/µL of each bacteria. For the analysis, we inferred a low concentration of bacteria between 2 and 2x10^2^ copies/µL and as high those between 2X10^3^ and 2X10^5^ copies/µL.

*Statistical analysis*

Different variables were evaluated for comparisons between hrHPV positive and negative findings for analytical calculations, and between NILM and LSIL women. A two-sided Fisher’s exact test for 2×2 contingency table was performed to evaluate the statistical significance between different groups. Crude odds ratios (OR, relative risk) with 95% confidence intervals (CI) were calculated to estimate the association of HPV and mycoplasmas positive findings with socio-demographic data. The age was expressed as a mean and standard deviation (SD). Statistical significance was defined as P values <0.05. All statistical analyses were performed using GraphPad Prism 6.0 (San Diego, California, USA).

## Results

*Baseline features*

Table 1 outlines the baseline features of the study cohort. A total of 360 women were attended during the study period for cervical cancer screening. Of these, 283 women fit the inclusion criteria of the study and were included with a mean (SD) age of 45.84 ± 11.04 years old (range 25-64). Most women exhibited the following characteristics: between 31-50 years old (51.2%); were married (68.2%); had education level ≥8 years (61.1%); were white (55.1%); and had 1-2 children (50.5%). 

A total of 273 (96.5%) women presented normal cytology (NILM) and 10 (3.5%) presented LSIL (Table 2). Women with NILM and LSIL had similar ages (45.90 ± 10.99 and 44.10 ± 12.84 years old, respectively; P = 0.30).

*hrHPV positivity*

Thirty-six women (12.7%) were positive for hrHPV (Tables 2 and 3). HPV16 was detected in 4 (11.1%) and HPV18 in 2 (5.6%) women. In the remaining 30 (83.3%) women, other types of hrHPV were detected. Twenty nine (10.6%) women with NILM and all (100%) women with LSIL were hrHPV positive. Thus, hrHPV was associated with LSIL (P < 0.0001) (Table 2). 

hrHPV positive women were younger than hrHPV negative women (41.42 ±10.52 vs. 46.49 ±10.99, respectively, p-value: 0.004) and most (51.2%) had between 31-40 years (P = 0.01) (Table 1).

*Cervical mycoplasmas positivity and quantification*

The PCR results for cervical mycoplasmas presented high rates of these bacteria (n = 152/283, 53.7%), with 142/283 (50.2%) positive women for *Ureaplasma* spp. and 61 (21.6%) for *Mycoplasma* spp. (Tables 2 and 3). In 51/152 of the women positive for mycoplasmas (33.6%). *Ureaplasma* spp. and *Mycoplasma* spp. were detected simultaneously (OR = 7.34, 95%CI = 3.36-16.00, P < 0.0001). For* Mycoplasma* spp. (n = 61), all samples (100%) were positive for *M. hominis* and none positive for M. genitalium. 

Women positive for *Ureaplasma* spp. had similar mean age to negative women (44.84 ± 10.65 vs. 46.85 ± 11.37 respectively, P = 0.07). Women positive for *M. hominis *also had similar mean age to negative women (45.05 ± 11.09 vs. 46.06 ± 11.04 respectively, P = 0.26). Women positive for *M. hominis* were associated with no children and with ≥3 children (P = 0.04 for both).

By rt-PCR, the samples of *Ureaplasma* spp. (n = 142) were identified as *U. parvum* (n = 125, 88%) and *U. urealyticum* (n = 17, 12%). None of the women was positive to both bacteria. Thus, *U. parvum* (n = 125/152, 82.2%) was the most frequently bacteria detected, followed by M. hominis (n = 61/152, 40.1%) and U. urealyticum (n = 17/152, 11.2%).

Regarding cytological findings, women positive for *U. parvum* have a 5-fold increased risk of LSIL (OR = 5.33; 95% CI = 1.09-26.04, P = 0.02) (Table 2).

**Figure 1:**
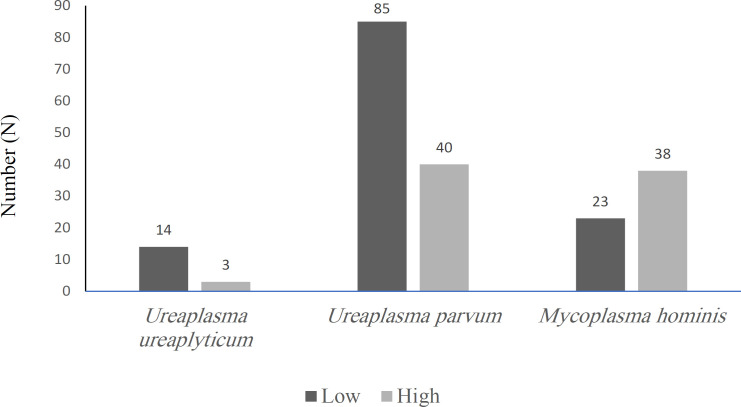
Quantification of Mycoplasmas in Copies/µL by rt-PCR as Low (between 2 and 2x10^2^ copies/µL) and high (between 2X10^3^ and 2X10^5^ copies/µL) Concentration

Figure 1 represents the quantification of mycoplasmas in copies/µL by rt-PCR as low and high concentration. It was possible to observe that despite *U. parvum* being the most detected mycoplasma it was quantified mainly in low concentrations (n = 85/125, 68%). On the other hand, the second most prevalent bacteria (*M. hominis*) was found mainly in higher concentrations (n = 38/61, 62.3%). *U. urealyticum* was quantified mainly in low concentrations (n = 14/17, 82.4%). 

*hrHPV and mycoplasmas co-infections*

Women positive for *U. parvum* had a 4-fold increased risk of hrHPV positivity (OR = 3.88; 95% CI = 1.75-8.58, P = 0.0003) (Table 3). Although we have evidenced the association between *U. parvum* with hrHPV, these associations were not dependent on the concentration of the bacteria, as shown in Table 4.

**Table 1:** Overall Baseline Features of the Study Cohort

Characteristics	Overall (n=283) n (%)	hrHPV+ (n=36) n (%)	hrHPV- (N=247) n (%)	OR (95%CI)	P value	*Ureaplasma* spp. + (n=142) n( %)	*Ureaplasma* spp. - (n=141) n (%)	OR (95%CI)	P value	*Mycoplasma hominis+ *(n=61) n (%)	*Mycoplasma hominis-*(n=222) n (%)	OR (95%CI)	P value
Age groups													
≤30 years	33 (11.7)	5 (13.9)	28 (11.34)	2.47 (0.71-8.52)	0.13	16 (11.3)	17 (12.1)	1.09 (0.49-2.41)	0.81	8 (13.1)	25 (11.3)	1.19 (0.47-3.01)	0.70
31-40 years	60 (21.2)	12 (33.3)	48 (19.43)	3.46 (1.25-9.58)	0.01	37 (26.0)	23 (16.3)	1.87 (0.97-3.62)	0.06	16 (26.2)	44 (19.8)	1.35 (0.64-2.85)	0.42
41-50 years	85 (30.0)	12 (33.3)	73 (29.55)	2.28 (0.84-6.13)	0.09	40 (28.2)	45 (31.9)	1.03 (0.58-1.84)	0.90	15 (24.6)	70 (31.5)	0.79 (0.38-1.66)	0.54
>50 years	104 (36.8)	7 (19.4)	97 (39.27)	1	-	48 (33.8)	56 (39.7)	1	-	22 (36.1)	82 (36.9)	1	-
No related	1 (0.3)	0 (0.0)	1 (0.40)	-	-	1 (0.7)	0 (0.0)	-	-	0 (0.0)	1 (0.5)	-	-
Marital status													
Married	193 (68.2)	20 (55.6)	173 (70.0)	1	-	93 (65.5)	100 (70.9)	1	-	36 (59.0)	157 (70.7)	1	
Unmarried	87 (30.7)	15 (41.7)	72 (29.2)	1.80 (0.87-3.73)	0.11	47 (33.1)	40 (28.4)	1.26 (0.76-2.10)	0.37	25 (41.0)	62 (27.9)	1.75 (0.97-3.18)	0.06
No related	3 (1.1)	1 (2.7)	2 (0.8)	-	-	2 (1.4)	1 (0.7)	-	-	0 (0.0)	3 (1.4)	-	
Education level													
<8 years	101 (35.7)	11 (30.6)	90 (36.4)	1.19 (0.55-2.57)	0.65	55 (38.7)	46 (32.6)	1.29 (0.79-2.12)	0.30	21 (34.4)	80 (36.0)	1.03 (0.56-1.89)	0.90
≥8 years	173 (61.1)	22 (61.1)	151 (61.1)	1	-	83 (58.5)	90 (63.8)	1	-	37 (60.7)	136 (61.3)	1	-
No related	9 (3.2)	3 (8.3)	6 (2.5)	-	-	4 (2.8)	5 (3.6)	-	-	3 (4.9)	6 (2.7)	-	-
Ethnic group													
White	156 (55.1)	18 (50.0)	138 (55.9)	1	-	78 (54.9)	78 (55.3)	1		32 (52.5)	124 (55.9)	1	-
No white	118 (41.7)	15 (41.7)	103 (41.7)	1.11 (0.53-2.32)	0.77	59 (41.6)	59 (41.9)	1		28 (45.9)	90 (40.5)	1.20 (0.67-2.14)	0.52
No related	9 (3.2)	3 (8.3)	6 (2.4)	-	-	5 (3.5)	4 (2.8)	-	-	1 (1.6)	8 (3.6)	-	-
Number of children													
0	25 (8.8)	4 (11.1)	21 (8.5)	1.32 (0.40-4.31)	0.64	12 (8.5)	13 (9.2)	1.11 (0.47-2.60)	0.81	8 (13.1)	17 (7.7)	2.59 (0.98-6.82)	0.04
1 or 2	143 (50.5)	18 (50.0)	125 (50.6)	1	-	65 (45.8)	78 (55.3)	1	-	22 (36.1)	121 (54.5)	1	-
≥3	93 (32.9)	10 (27.8)	83 (33.6)	0.83 (0.37-1.90)	0.67	52 (36.6)	41 (29.1)	1.52 (0.89-2.58)	0.11	24 (39.3)	69 (31.1)	1.91 (0.99-3.68)	0.04
No related	22 (7.8)	4 (11.1)	18 (7.3)	-	-	13 (9.1)	9 (6.4)	-	-	7 (11.5)	15 (6.7)	-	-

hrHPV, high-risk human papilomavirus

**Table 2:** Association between hrHPV and Mycoplasmas with Cytological Findings

	Overall (n = 283) n (%)	Normal (n = 273) n (%)	LSIL (n = 10) n (%)	OR (95%CI)	P -value
hrHPV					
Positive	3 (12.7)	26 (9.5)	10 (100.0)	-	< 0.0001
Negative	247 (87.3)	247 (90.5)	0 (0.0)	1	
*Ureaplasma parvum*					
Positive	125 (44.2)	117 (42.9)	8 (80.0)	5.33 (1.09-26.04)	0.02
Negative	158 (55.8)	156 (57.1)	2 (20.0)	1	
*Ureaplasma urealyticum*					
Positive	17 (6.0)	17 (6.3)	0 (0.0)	-	> 0.99
Negative	266 (94.0)	256 (93.7)	10 (100.0)	1	
*Mycoplasma hominis*					
Positive	61 (21.5)	59 (21.6)	2 (20.0)	1.10 (0.26-5.27)	> 0.99
Negative	222 (78.5)	214 (78.4)	8 (80.0)	1	

hrHPV, high-risk human papillomavirus; LSIL, low-grade squamous intraepithelial lesions

**Table 3:** Overall Rates of hrHPV and Mycoplasmas co-Infections in Women Included in the Study

Mycoplasmas	Overall (n=283) n (%)	hrHPV + (n=36) n (%)	hrHPV – (n=247) n (%)	OR (95%CI)	P- value
*Ureaplasma parvum*					
Positive	125 (44.2)	26 (72.2)	99 (40.1)	3.88 (1.75-8.58)	0.0003
Negative	158 (55.8)	10 (27.8)	148 (59.9)	1	
*Ureaplasma urealyticum*					
Positive	17 (6.0)	2 (5.6)	15 (6.1)	0.91 (0.19-4.16)	0.90
Negative	266 (94.0)	34 (94.4)	232 (93.9)	1	
*Mycoplasma hominis*					
Positive	61 (21.6)	8 (22.2)	53 (21.5)	1.04 (0.45-2.43)	0.92
Negative	222 (78.4)	28 (77.8)	194 (78.5)	1	

hrHPV, high-risk human papillomavirus

**Table 4:** Rates of hrHPV and *U. parvum* co-Infections in Relation to Bacteria Concentrations

*U. parvum* concentration	hrHPV+ (n=26) n (%)	hrHPV- (n=99) n (%)	OR (95%CI)	P value
Low	23 (88.5)	92 (92.9)	1	-
High	3 (11.5)	7 (7.1)	1.71 (0.45-7.07)	0.43

hrHPV, high-risk human papillomavirus; Low, between 2 and 2x10^2^ copies/µL of *U. parvum*; High, between 2X10^3^ and 2X10^5^ copies/µL of U. parvum.

## Discussion

This study investigated the rates of co-infections between hrHPV and important human mycoplasmas in cervical samples of asymptomatic Brazilian population. We found that *U. parvum* was the primary mycoplasma associated with hrHPV for increasing the risk of LSIL in this population. Therefore, *U. parvum* might play a synergistic role in the initial stage of cervical lesions caused by hrHPV infection. 

Previous reports presented high rates of ureaplasmas and *M. hominis* detection in asymptomatic sexually mature females (Taylor-Robinson et al., 2017). Epidemiological data showed that *Ureaplasma* spp. can be detected in the cervix or vagina of 40-80% of sexually mature asymptomatic females, whereas *M. hominis* and M. genitalium only in 20-50% and 0-5%, respectively (Morse et al., 2010). Our results are consistent with these evidences since *Ureaplasma* spp. was detected in 142 (50.2%) women, *M. hominis* in 61 (21.6%), and *M. genitalium* was not detected. Additionally, we found that *U. parvum* was the most frequent bacterial agent detected (n = 125/283, 44.2%). Our results are similar to obtained in a recent study, which *U. parvum* was the most prevalent mycoplasma in the cervix (Carneiro et al., 2020). However, for these authors, *U. parvum*, *M. hominis* and *U. urealyticum* were detected at much lower rates (14.9%, 8.5% and 4.2%, respectively) than in our study, and *M. genitalium *was also not detected. Foschi et al., (2018) also found that *U. parvum* was the most prevalent mycoplasma (67.3%), followed by *M. hominis* (14.3%), U. urealyticum (9.2%) and *M. genitalium* (3.1%) in the group of asymptomatic healthy women. 

HPV is necessary, but not sufficient, to cause cervical cancer, which supports the influence of additional factors in carcinogenesis associated with hrHPV (Ho et al., 1998; Kjaer et al., 2010; Tota et al., 2011; Ko et al., 2015; Monsonego et al., 2015). Mycoplasmas have been suggested as a possible risk factor for the development of cervical lesions (Ekiel et al., 2009) and cervical cancer (Biernat-Sudolska et al., 2011), and with HPV infections in samples with abnormal cytology (Biernat-Sudolska et al., 2011; Kawaguchi et al., 2012). Our results are in agreement with these findings once women positive for *U. parvum* showed a 5-fold increased risk of LSIL. Additionally, we observed significant association between *U. parvum* and hrHPV infections, indicating that women with both infections might be more likely to develop cervical lesions. Our results are consistent with the hypothesis that mycoplasmas can exert a synergistic action with hrHPV and possibly act as cofactors in the development of cervical lesions (Ho et al., 1998; Bodily et al., 2011; Tota et al., 2011). Therefore, detecting and eradicating *U. parvum* infection might contribute to reduce HPV-related carcinogenesis (Drago et al., 2016).

However, our results also suggest that there exist differences between different mycoplasmas regarding the potential to act as a cofactor for hrHPV since only *U. parvum* was associated with LSIL and hrHPV. These results are in agreement with Drago et al., (2016) which found a strong correlation between *U. parvum*-HPV co-infection and CIN1. According to these authors, this data indicates that *U. parvum* may be involved in HPV persistence and in promoting the development of cytological abnormalities. Additionally, in a recent meta-analysis, *U. parvum* was also associated with a significantly increased risk of abnormal cervical cytopathology (Ye et al., 2018). Finally, Camporiondo et al., (2016) showed that the probability of HPV-positive women being infected by *U. parvum* was 2.5 fold higher than in HPV-negative women.

The association between *U. urealyticum* and *M. hominis* with abnormal cervical cytopathology and with hrHPV was not observed in our results, in contrary to other authors (Magaña-Contreras et al., 2015; de Abreu et al., 2016; Kim et al., 2016; Kim et al., 2018; Klein et al., 2020). This can be explained, at least in part, by the differences between the populations evaluated in the different studies as well as in the techniques used to detect and identify mycoplasmas. Thus, it is very important that future studies should be carried out in different populations with similar clinical conditions to elucidate the participation of different mycoplasmas as cofactors of hrHPV in cervical carcinogenesis.

Although we have evidenced the association between *U. parvum* with LSIL and with hrHPV, these associations were not dependent on the concentration of the bacteria. This data may indicate that possibly the presence of the bacteria regardless of their concentration in the cervical environment would be sufficient to act synergistically with hrHPV in the development of cervical lesions. In a recent study, Klein et al., found different results regarding the mycoplasmas concentration and cervical lesions. More specifically, these authors showed that *M. hominis *prevalence was similar despite the severity of cervical lesions; however the abundance of *M. hominis* increased significantly in women with cervical lesions. Therefore, the role of *U. parvum* concentration as a risk factor for the development of cervical lesions has not been completely elucidated and requires further investigation.

The present study had no data on the history of hrHPV and vaginal infections as well as mycoplasmas agents in the women prior to enrollment in the study or during follow-up, which limits our interpretations on the influence of hrHPV persistence. As we evaluated mycoplasmas and hrHPV infections at the same time, we were not able to rule out reverse causation. It is important to note that our study did not aim to assess the carcinogenicity mechanisms of the synergistic action between hrHPV and mycoplasmas agents, but assessed the possible occurrence of this synergistic action. 

In conclusion, this study provided an opportunity to determine the rates of co-infection between hrHPV and important mycoplasmas in different cytological cervical samples. We found that *U. parvum* was the primary pathogen associated with hrHPV for the increased risk of cervical abnormalities, particularly for LSIL in an asymptomatic population. Our results reinforce the hypothesis that some mycoplasmas may play a role as cofactors in hrHPV-mediated cervical carcinogenesis. Further cross-sectional studies are needed in different populations mainly in those with high rates of HSIL and cervical cancer.

## Author Contribution Statement

IHY Noma, CS Shinobu-Mesquita, TT Suehiro, MEL Consolaro, and F Morelli designed and wrote the study protocol; IHYNoma, MVF de Souza, and E Damke were in charge of HPV analysis; IHY Noma, VRS da Silva, and CS Shinobu-Mesquita managed the data and performed the statistical analysis; and IHY Noma, CS Shinobu-Mesquita, TT Suehiro, F Morelli, MVF de Souza, E Damke, VRS da Silva, and MEL Consolaro wrote the manuscript. All of the authors have read and approved the final manuscript.

## Funding Statement

This study was supported by the Coordenação de Aperfeiçoamento de Pessoal de Nível superior (CAPES), Brazilian Government.

Approvation by scientific Body/part of an student thesis: This study was part of an approved student thesis (Isabella Harumi Yonehara Noma).

## Ethics approval and consent to participate

Prior to undergoing gynecological examination, all women signed a written informed consent. Each woman voluntarily agreed to provide a sample of uterine cervix for Pap screening, hrHPV test and molecular mycoplasmas detection. This study was conducted with the supervision and approval of the Committee for Ethics in Research Involving Humans at the State University of Maringá (UEM)/Paraná/Brazil (CAAE: 37091114.2.0000.0104).
